# A comprehensive cotton leaf disease dataset for enhanced detection and classification

**DOI:** 10.1016/j.dib.2024.110913

**Published:** 2024-09-10

**Authors:** Prayma Bishshash, Asraful Sharker Nirob, Habibur Shikder, Afjal Hossan Sarower, Touhid Bhuiyan, Sheak Rashed Haider Noori

**Affiliations:** Department of Computer Science and Engineering, Daffodil International University, Daffodil Smart City, Birulia, Dhaka 1216, Bangladesh

**Keywords:** Agricultural dataset, Machine learning in agriculture, Precision farming, Deep learning for crop management, Smart farming

## Abstract

The creation and use of a comprehensive cotton leaf disease dataset offer significant benefits in agricultural research, precision farming, and disease management. This dataset enables the development of accurate machine learning models for early disease detection, reducing manual inspections and facilitating timely interventions. It serves as a benchmark for testing algorithms and training deep learning models, aiding in automated monitoring and decision support tools in precision agriculture. This leads to targeted interventions, reduced chemical use, and improved crop management. Global collaboration is fostered, contributing to the development of disease-resistant cotton varieties and effective management strategies, ultimately reducing economic losses and promoting sustainable farming. Field surveys conducted from October 2023 to January 2024 ensured meticulous image capture under diverse conditions. The images are categorized into eight classes, representing specific disease manifestations, pests, or environmental stress in cotton plants. The dataset comprises 2137 original images and 7000 augmented images, enhancing deep learning model training. The Inception V3 model demonstrated high performance, with an overall accuracy of 96.03 %. This underscores the dataset's potential in advancing automated disease detection in cotton agriculture.

Specifications Table

**Table:** 

Subject	Computer Science.
Specific subject area	Classification of cotton leaf, Diseases Image Classification, Image Recognition, Deep Learning, and computer vision.
Type of data	Image.
Data collection	Field surveys conducted from October 2023 to January 2024, supervised by experts, ensured meticulous image capture under different environmental conditions. The dataset comprises a collection of 2137 images depicting various stages of cotton leaf diseases. These images are categorized into eight classes: bacterial blight, curl virus, herbicide growth damage, leaf hopper jasisds, leaf reddening, leaf variegation, and healthy leaves. Each class represents specific manifestations of diseases, pests, or environmental stress in cotton plants. This dataset provides a comprehensive range of visual traits essential for training and evaluating machine learning models aimed at accurately classifying and diagnosing cotton leaf diseases.
Data source location	The National Cotton Research Institute field in Gazipur, Dhaka, Bangladesh
Data accessibility	Repository name: Mendeley Data Data identification number: 10.17632/b3jy2p6k8w.2 Direct URL to data: https://data.mendeley.com/datasets/b3jy2p6k8w/2
Related research article	None

## 1 Value of the Data

• The presence of diseases such as Cotton Leaf Curl Disease and leaf hopper in cotton plants poses significant challenges to farmers worldwide, leading to substantial yield losses, reduced crop quality, and economic hardships. Timely detection and effective management of these diseases are critical for sustaining cotton production and ensuring food security. Therefore, identifying plant diseases at the early stage will benefit in diagnosing and preventing unnecessary crop loss. Among different parts of the plant, the leaf is the part that affects the crop yield if it gets affected. Visible symptoms can help in the detection of disease, and plant pathologists can suggest a suitable pesticide [1]. Traditional methods of disease detection often rely on manual inspection by agronomists, which can be time-consuming, subjective, and prone to human error. By contrast, utilizing computer vision and machine learning techniques with the aid of a comprehensive cotton leaf disease dataset can revolutionize disease detection and management practices.
• The availability of high-quality, labeled images of cotton plants affected by various diseases enables the development and training of sophisticated algorithms for automated disease identification and classification. Such systems can accurately detect the presence of diseases, distinguish between different types of infections, and quantify disease severity, thereby facilitating prompt intervention measures. According to experts, the most common approach to identify the diseases is the naked eye approach. This approach has many limitations, such as the requirement for continuous monitoring by experts, which may be expensive and time-consuming, particularly in vast farms and remote areas [2]. Using traditional approaches, it is almost impossible to identify diseases at an early stage with good accuracy and minimal computational time. Therefore, it is important to formulate methods of disease identification that can perform the task automatically, accurately, timely, and inexpensively [3]. With the aid of computer vision algorithms trained on the cotton leaf disease dataset, farmers can implement precision agriculture techniques, such as targeted pesticide application and selective breeding of disease-resistant cultivars. This targeted approach not only minimizes the use of agrochemicals, reducing environmental impact, but also optimizes resource utilization and enhances overall crop health.
• The cotton leaf disease dataset serves as a valuable resource not only for researchers in the fields of computer vision and machine learning but also for agricultural scientists and policymakers. Collaborative efforts between these disciplines can lead to the development of integrated disease management strategies, informed by data-driven insights and tailored to local agro ecological conditions. By empowering farmers with advanced tools for disease detection and management, the cotton leaf disease dataset contributes to sustainable agricultural practices, resilience against emerging threats, and socioeconomic development in cotton-growing regions. Ultimately, its widespread adoption can lead to increased productivity, profitability, and resilience in the cotton industry, benefitting stakeholders across the entire value chain.

## 2 Background

This dataset comprises a collection of 2137 original images showcasing various stages of cotton leaf diseases, including bacterial bright, curl virus, herbicide growth damage, Leaf hopper jassids, healthy leaf, leaf redding and, leaf variegation. Each image vividly illustrates characteristic symptoms such as leaf discoloration, curling, wilting, necrosis, and other discernible features associated with these diseases. These images are meticulously captured under diverse environmental conditions and at different growth stages of cotton plants, ensuring a comprehensive representation of disease manifestations. This dataset serves as a critical resource for researchers and practitioners in the realm of precision agriculture. By leveraging these images, they can develop and validate sophisticated algorithms aimed at accurately detecting and classifying cotton leaf diseases. These algorithms play a pivotal role in disease management strategies, enabling farmers to identify and address diseases promptly, thereby minimizing crop losses and optimizing yield potential. Furthermore, the availability of this dataset fosters collaboration and innovation within the agricultural community. Researchers can leverage this rich repository of images to explore novel approaches for disease detection and management, while practitioners can integrate these technologies into their farming practices to enhance overall crop health and productivity. Ultimately, this dataset contributes to the advancement of sustainable agriculture practices, promoting resilience and prosperity in cotton production systems worldwide.

## 3 Data Description

The dataset comprises a collection of images depicting various stages of cotton leaf diseases, sourced from the National Cotton Research Institute field in Gazipur. The images were captured using a Redmi Note 11s smartphone. The resultant images exhibit three different dimensions: 3000×4000 pixels, 2239×2239 pixels, and 1597×1597 pixels, ensuring a diverse representation of disease manifestations. These images were meticulously taken during field surveys conducted at different time intervals, spanning from October 2023 to January 2024, under the guidance of domain experts. Additionally, images were captured under different environmental conditions to encapsulate the natural variability observed in cotton cultivation.

Despite challenges encountered during data collection, such as fluctuating lighting conditions and potential background noise, efforts were made to ensure high-quality image acquisition. The primary focus was on capturing images that accurately depict the characteristic symptoms associated with each cotton leaf disease class.

**Fig. 1:**
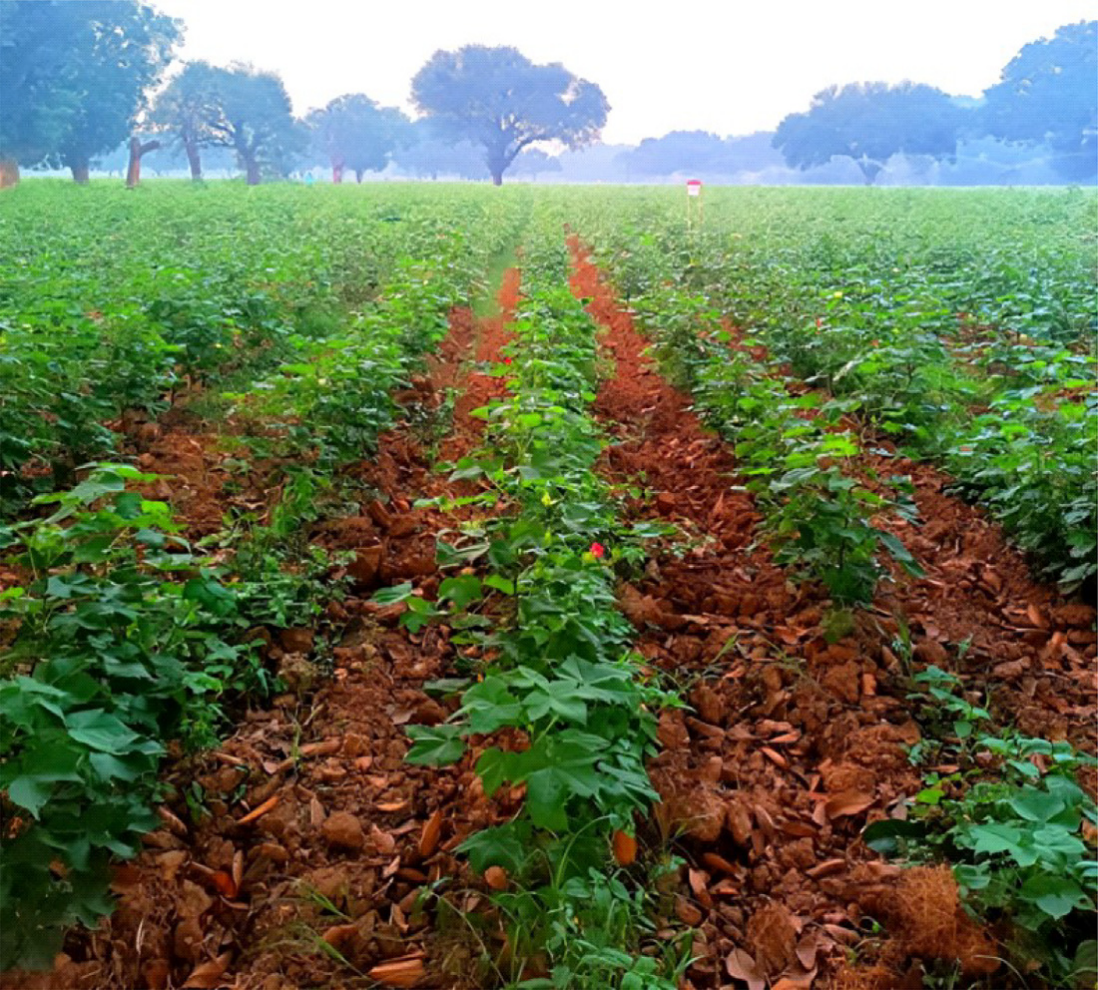
The real cotton field from where dataset images were gathered.

Fig. 1 showcases the natural environment where cotton plants were observed and photographed, providing context for the dataset's origin and the real-world conditions under which the images were captured.

Identifying the varieties of cotton leaf diseases holds significant importance for effective disease management in agriculture. This process enables targeted treatment strategies, as different disease varieties may require specific interventions such as tailored pesticide or fungicide applications. Understanding prevalent disease varieties also facilitates the implementation of preventive measures, allowing farmers to take proactive steps to mitigate disease risks based on environmental conditions and crop growth stages [4]. Also, research efforts benefit from the classification of disease varieties, contributing to the development of new disease-resistant crop varieties and innovative management strategies, ultimately enhancing agricultural productivity and food security. For that reason, we have presented two varieties of cotton: American upland and CB-12 to CB-18 (Table 1).

**Table 1:** Details about the cotton varieties in the dataset.

Variety Name	Description
American Upland	American Upland cotton, scientifically known as Gossypium hirsutum, is one of the most widely cultivated and economically significant cotton species globally. It is valued for its fiber quality, yield potential, and adaptability to various growing conditions. American Upland cotton accounts for the majority of cotton production worldwide and is extensively used in the textile industry for producing a wide range of products, including clothing, bedding, and industrial textiles [5]. Its resilience to pests and diseases, coupled with advancements in breeding and agricultural practices, has contributed to its widespread cultivation and commercial success.
CB-12 to CB-18	CB-12 and CB-13 exhibit 40 bolls per plant, with CB-13 having slightly higher boll weight and seed cotton yield. CB-14 distinguishes itself with 45 bolls per plant and a moderate seed cotton yield. CB-15 boasts the tallest plant height and highest seed cotton yield among the varieties. CB-16 and CB-17 demonstrate similarities in bolls per plant and seed cotton yield, with CB-17 showcasing a higher percentage of ginning outturn [6]. This information serves as valuable guidance for farmers and researchers in selecting appropriate cotton varieties based on factors such as yield potential and growth cycle duration.

In our dataset, we compiled images representing eight categories of cotton leaf conditions: bacterial blight, curl virus, herbicide growth damage, leaf hopper jasisds, leaf reddening, leaf variegation, and healthy leaves (see in Table 2). Each category usually corresponds to a specific manifestation of disease, pest or environmental stress in the cotton plant. The dataset covers a wide range of visual traits including discoloration, deformation, lesions and pest infestation observed in cotton plants at different stages of growth. These images serve as valuable resources for training and evaluating machine learning models aimed at accurately classifying and diagnosing cotton leaf diseases and abnormalities.

**Table 2:** Cotton Leaf Class wise disease overview.

Class Name	Description	Visualization
Bacterial Blight	It is a common disease affecting cotton plants caused by the bacterium Xanthomonas citri pv. malvacearum. This bacterial pathogen primarily targets the leaves of cotton plants, leading to characteristic symptoms such as angular water-soaked lesions that later turn brown or black. The affected areas may also exhibit tissue necrosis and leaf drop, ultimately impacting the plant's photosynthetic capacity and overall health [7]. Bacterial Blight can spread rapidly under favorable environmental conditions, including high humidity and warm temperatures, posing a significant threat to cotton crops and necessitating prompt management strategies to mitigate its impact on yield and quality.	
Curl Virus	Cotton leaf curl virus (CLCuV) is a destructive pathogen that infects cotton plants, causing significant economic losses in cotton-growing regions worldwide. This virus is transmitted by whiteflies and belongs to the genus Begomovirus. Symptoms of cotton leaf curl virus infection include leaf curling, vein yellowing, stunted growth, and reduced yield. Infected plants may also exhibit leaf crumpling, puckering, and distortion. CLCuV can severely impact cotton production by reducing fiber quality and yield [8].	
Herbicide Growth Damage	Herbicide growth damage on cotton leaves occurs when herbicides are applied improperly or at incorrect dosages, leading to adverse effects on plant growth and development. Symptoms of herbicide damage on cotton leaves vary depending on the type of herbicide and the extent of exposure but commonly include leaf yellowing, necrosis, stunted growth, and leaf deformation [9]. Proper application techniques, including accurate calibration of equipment and adherence to recommended application rates and timing, are crucial for minimizing herbicide damage on cotton leaves and maintaining crop health and yield.	
Leaf Hopper Jassids	Leafhopper jassids, commonly referred to as leafhoppers, are small insects that feed on the sap of cotton plants, causing damage to the leaves. Infestations of leafhopper jassids can result in characteristic symptoms such as stippling, yellowing, and browning of leaves, which can lead to reduced photosynthetic activity and decreased plant vigor. Severe infestations may cause leaf curling or distortion, further impacting the plant's ability to grow and produce cotton [10]. Early detection and intervention are essential for minimizing damage and preserving cotton yield and quality.	
Leaf Reddening	Leaf reddening in cotton plants is a visual symptom characterized by the reddish or purplish discoloration of leaves, typically starting at the tips or edges and spreading towards the center and veins. This condition often indicates physiological stress caused by various factors such as nutrient deficiencies, particularly phosphorus, potassium, or magnesium, which disrupt chlorophyll synthesis and photosynthesis [11]. Managing nutrient levels, optimizing irrigation, and implementing pest and disease control measures are essential for addressing leaf reddening and ensuring healthy cotton plant growth.	
Leaf Variegation	Leaf variegation in cotton plants refers to irregular patterns of different colors on the leaves, caused by factors like genetic mutations, viral infections, nutrient imbalances, or herbicide damage. It can affect photosynthesis and nutrient absorption, leading to stunted growth and reduced productivity [12]. Proper diagnosis and management are essential for maintaining plant health and yield, involving adjustments in nutrient levels, pest control, environmental conditions, and cultivar selection.	
Healthy Leaf	Healthy cotton leaf present vibrant green color, uniform shape, and absence of disease or pest damage, crucial for photosynthesis and plant growth [13]. Monitoring leaf health aids early issue detection, ensuring optimal yield and quality through proper agricultural practices.	

In the field of agriculture science, automation has emerged as a game-changer, offering numerous benefits to a nation's agriculture economy. One of the primary advantages is the enhancement of product quality. Automation, particularly in tasks like fruit and vegetable sorting and grading, enables the production of uniform and high-quality results. This is essential for meeting the demands of customers and global markets, which often have stringent quality standards.

Manual processes can be slower and less accurate than automated systems, leading to reduced productivity and efficiency. Increased Labor Costs: Manual processes require more labor, leading to increased labor costs for the business [14]. To address these challenges, intelligent fruit grading systems have been developed. These systems utilize computer vision algorithms to automatically classify and evaluate products based on various quality criteria. Computer vision technology enables precise measurement and analysis of traits such as color, texture, size, shape, and flaws, providing accurate and objective assessments of product quality. By automating sorting and grading processes, these systems not only improve efficiency but also reduce the risk of errors and ensure consistency in product quality, ultimately benefiting farmers, producers, and consumers alike [15].

The Cotton Leaf Dataset holds promise across various applications:

**Developing automated disease management systems**: The dataset is a key resource for developing automated systems to manage cotton leaf diseases. It enables the creation of machine learning models that accurately identify and classify diseases, allowing for early detection and intervention. These models help farmers and experts minimize crop losses and improve disease management through continuous refinement and real-world application.

**Improving precision agriculture practices**: The Cotton Leaf Dataset advances precision agriculture by enabling machine learning algorithms to analyze disease prevalence, severity, and distribution. This helps farmers make data-driven decisions on disease control, resource allocation, and crop management, leading to optimized yields and sustainable cotton production.

**Empowering remote sensing technologies**: The dataset supports the development of remote sensing technologies for monitoring cotton leaf health and disease dynamics. By using image analysis algorithms, UAVs and satellite systems can detect subtle changes in leaf health and identify disease hotspots. This enables timely intervention, targeted management, and improved crop resilience and productivity.

The Cotton Leaf Disease Dataset stands out by addressing key gaps in existing datasets through its field-sourced, high-resolution images that capture real-world disease manifestations under diverse environmental conditions. Unlike datasets limited to a few disease categories or those captured in controlled environments, this dataset offers a comprehensive representation of cotton leaf diseases, including bacterial blight, curl virus, and herbicide damage, among others. Its inclusion of different cotton varieties, such as American Upland and CB-12 to CB-18, adds further depth, enabling the exploration of disease responses across different types. The dataset's novelty lies in its ability to support the development of robust machine-learning models for precision agriculture, disease management, and remote sensing applications, making it a valuable resource for advancing sustainable cotton farming practices.

## 4 Experimental Design, Materials and Methods

### 4.1 Experimental Design

**Fig. 2:**
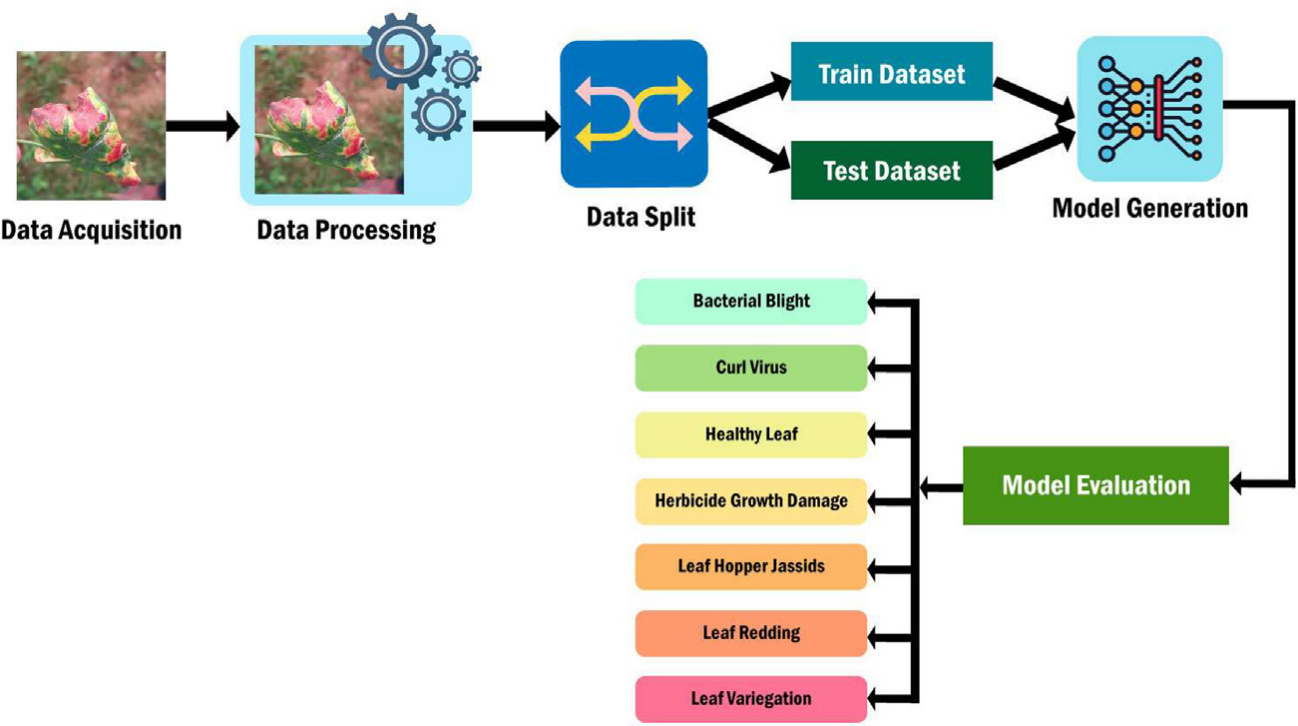
The method by which the diseases of cotton leaf is evaluated.

The dataset utilized in this study was sourced from the National Cotton Research Institute field in Gazipur, covering the period from October 2023 to January 2024. Data collection involved the compilation of images depicting cotton leaves, forming the foundational dataset for training and assessing the machine learning model. Following data acquisition, preprocessing steps were applied to the leaf images, including resizing, normalization, and cleaning, to optimize their suitability for model training. Subsequently, the dataset was partitioned into two subsets: 90 % training dataset and 10 % valid dataset. The former was employed to train the machine learning model, while the latter was dedicated to evaluating its performance. Machine learning algorithms, particularly deep learning models, were leveraged to develop a model capable of identifying various leaf conditions, encompassing bacterial bright, curl virus, herbicide growth damage, Leaf hopper jassids, healthy leaf, leaf redding and, leaf variegation. Model performance was assessed using the test dataset, with metrics such as accuracy, precision, and recall utilized to measure its efficacy in predicting leaf conditions. The process of employing machine learning techniques to enhance agricultural practices pertaining to cotton leaf health is elucidated in Fig. 2.

To propel progress in cotton research, we introduce the Cotton Leaf Disease Detection Dataset, a valuable resource for advancing disease detection in cotton plants. This dataset is structured into two distinct subfolders: the original dataset, consisting of 258MB of images captured directly from a camera, and the augmented dataset, which includes 1.20GB of images generated through data augmentation methods. Occupying a total space of 1.458GB, the Cotton Leaf Disease Detection Dataset encompasses a wide range of images reflecting various states of cotton leaf health. Attributes such as color, texture, size, and shape are intricately linked to the quality and well-being of cotton leaves. Our primary objective is to detect diseases promptly and accurately, thereby mitigating yield losses and safeguarding crop health.

### 4.2 Materials (Camera Specification)

Capturing the entirety of my data with the Redmi Note 11S, I've experienced its revolutionary camera capabilities first-hand. The 108 MP primary camera, coupled with an f/1.9 aperture, excels in capturing fine details, even in dimly lit conditions. Its 8 MP ultra-wide lens offers a broad 118° field of view, perfect for expansive landscapes. Additionally, the 2 MP macro lens highlights the beauty of close-up subjects, while the 2 MP depth sensor adds dimension to portraits. With this versatile setup, every shot is a testament to exceptional performance.

### 4.3 Methods

**Fig. 3:**
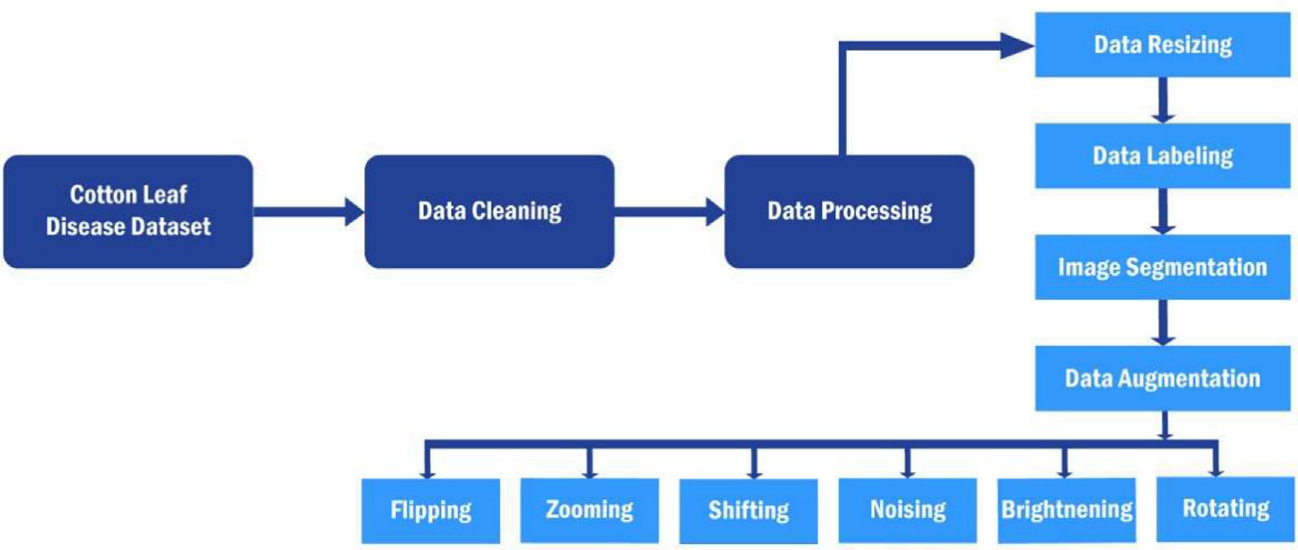
The proposed deep learning model's pre-processing stages.

We detail the comprehensive process of preparing and augmenting the Cotton Leaf Disease Dataset for machine learning and data analysis. We initiate the process by acquiring a comprehensive dataset containing images of cotton leaves affected by various diseases. Following this, we conduct meticulous data cleaning procedures to eliminate irrelevant or erroneous data, ensuring the quality of the dataset. Subsequently, we proceed with data processing, which involves standardizing the resolution of images, labeling each image with pertinent disease information, and employing advanced segmentation techniques for detailed analysis. Also, we employ data augmentation techniques, including flipping, zooming, shifting, noising, brightening, and rotating, to enhance dataset diversity and robustness. This rigorous approach ensures that the dataset is well-suited for training accurate and reliable machine learning models capable of effectively identifying and classifying various leaf conditions, thereby contributing to advancements in agricultural disease management and crop health monitoring. The systematic process outlined in Fig. 3 illustrates each step of dataset management, from initial data cleaning to final data augmentation, ensuring effective preparation for further analysis, particularly in machine learning or data science applications focused on recognizing and classifying diseases in cotton leaves.

### 4.4 Data Annotation Protocol

Data annotation was performed by trained experts familiar with cotton leaf diseases. Annotators followed a standardized protocol to label images with disease information. Conflicts were resolved through a consensus process, where discrepancies were reviewed by multiple annotators to ensure accurate labeling.

### 4.5 Data Augmentation

**Fig. 4:**
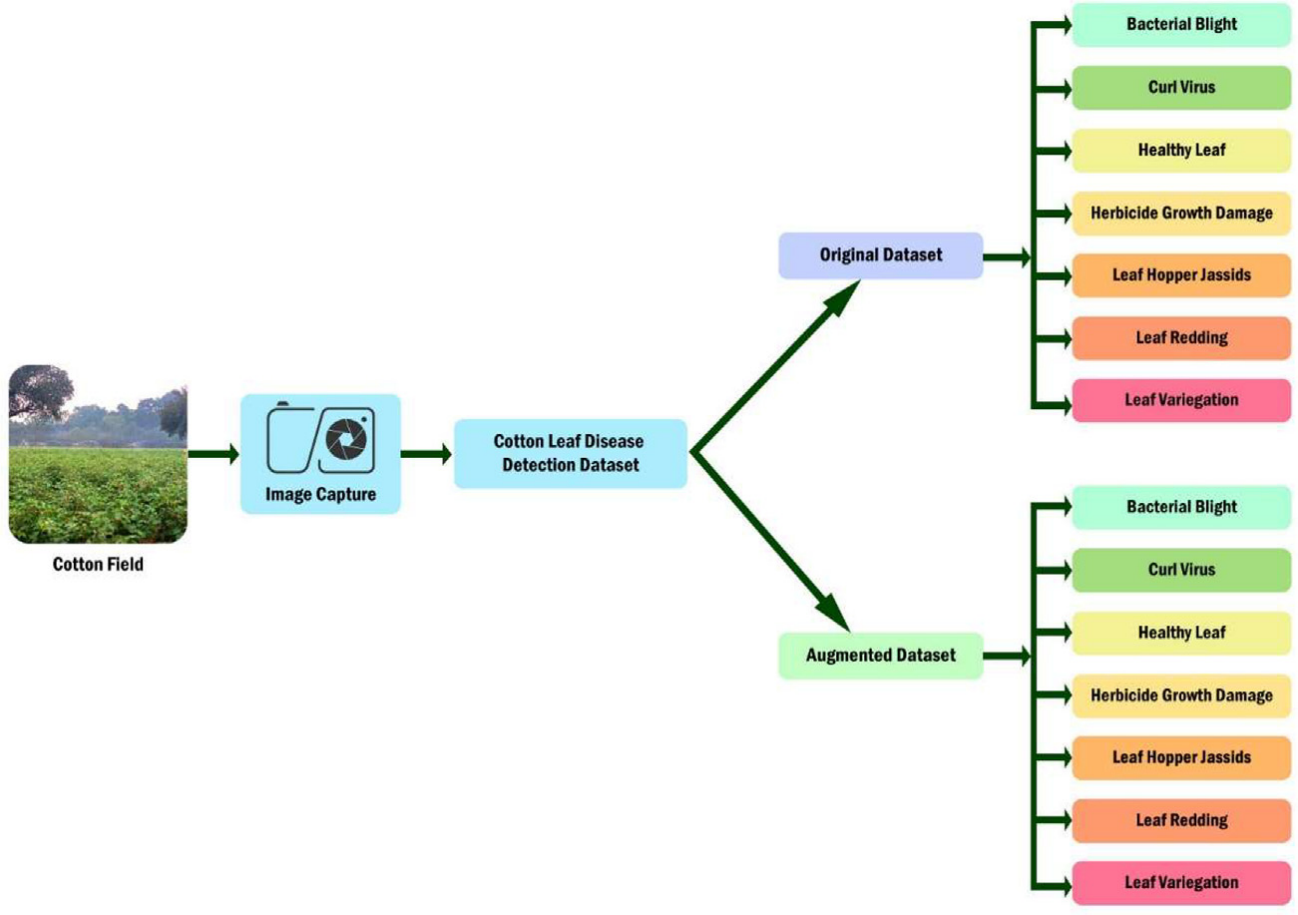
Dataset organization of cotton leaf disease.

The cotton dataset serves as a foundational resource in agricultural research, playing a pivotal role in advancing the development of sophisticated machine learning algorithms aimed at accurately classifying and detecting various diseases affecting the cotton crop. By leveraging dataset, researchers can harness the power of artificial intelligence to enhance agricultural productivity and bolster efforts in crop protection [16]. To construct this dataset, we meticulously collected images from cotton fields, capturing diverse manifestations of cotton leaf diseases under different environmental conditions. Subsequently, we partitioned the dataset into two integral branches: the original dataset and the augmented dataset. Each branch encompasses classifications representing distinct cotton leaf diseases and essential conditions crucial for effective disease detection and management. Noteworthy classifications include bacterial blight, curl virus, healthy leaves, herbicide growth damage, leaf hopper cysts, leaf redding, and leaf variegation, as depicted in Fig. 4. This dataset serves as a visual roadmap, delineating the structured process for cotton foliar disease detection, thereby catalyzing advancements in agricultural disease management and facilitating crop health monitoring initiatives.

Data augmentation is a crucial technique in machine learning, particularly in the realm of deep learning and computer vision. It involves generating new training samples by applying various transformations to existing data. By creating diverse versions of the original data, data augmentation helps prevent overfitting, improves model generalization, and enhances the robustness of machine learning models [17]. This technique is especially beneficial when working with limited datasets, as it effectively expands the available training data without the need for additional data collection. Overall, data augmentation plays a vital role in improving the performance and reliability of machine learning models across various applications and domains.

**Fig. 5:**
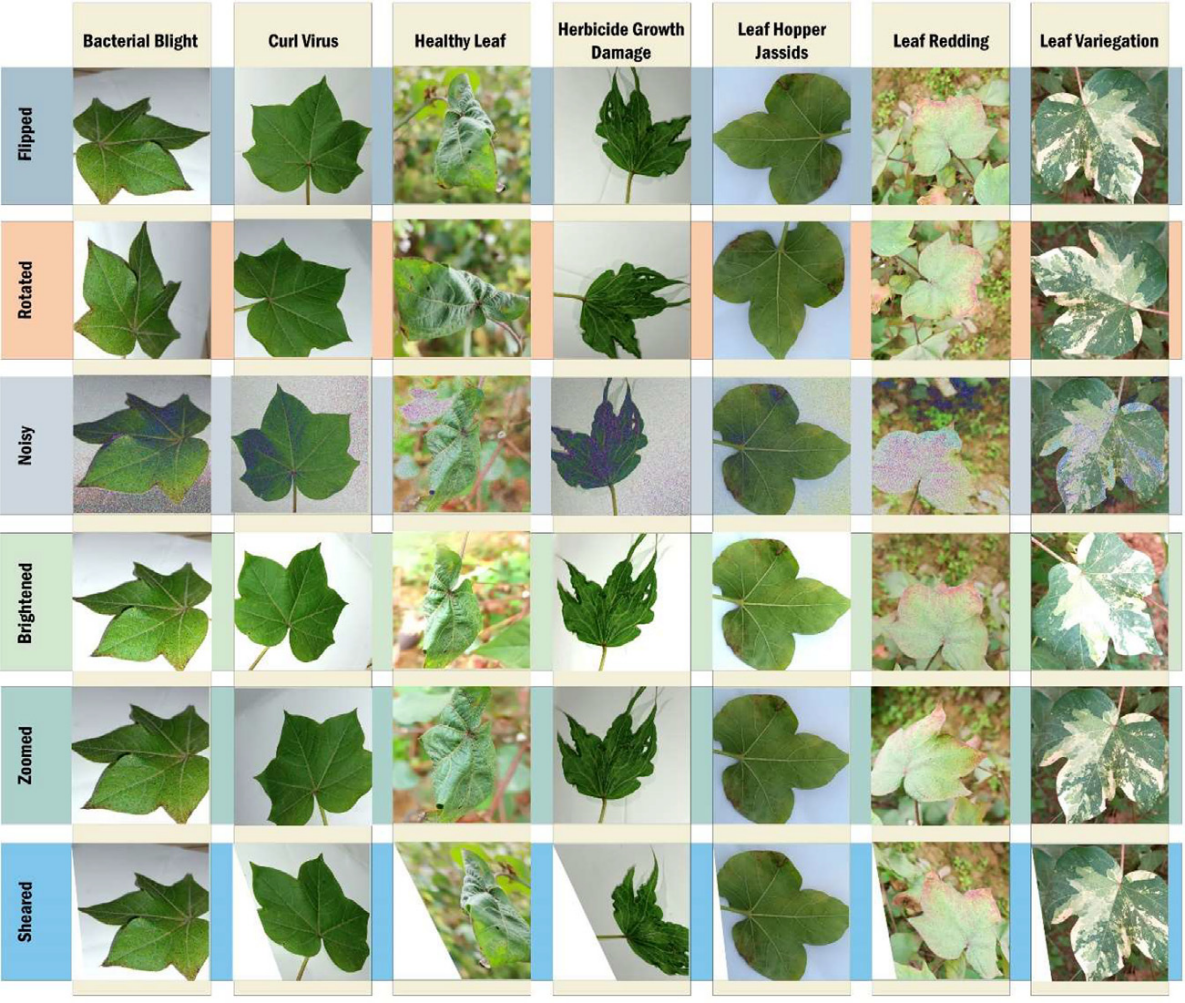
Cotton leaf disease dataset's augmented photos.

Data augmentation proves indispensable for refining deep learning models, particularly in visual object recognition tasks. By amplifying the training dataset with diverse image variations, it bolsters model generalization and mitigates overfitting. Our approach employs a range of augmentation techniques, including rescale, rotation range, width and height adjustments, shearing, zooming, horizontal and fill modifications, in line with established best practices. The augmentation parameters were carefully calibrated, with a rotation range of 45 degrees, width and height shift ranges of 0.3, a shear range of 0.3, and a zoom range of 0.3 to ensure dataset diversity. Horizontal flipping was applied, complemented by ‘reflect’ fill mode to manage modifications seamlessly. Brightness adjustments within the range of 0.5 to 1.5 were made to simulate varied lighting conditions (see in Fig. 5).

Through meticulous augmentation, we fortified our dataset, enriching the efficacy of deep learning model training. With 2137 original images and 7000 meticulously paired augmented images showcased in Table 3, our dataset exemplifies the profound impact of augmentation in enhancing and fortifying our dataset, ultimately leading to improved model performance.

**Table 3:** Statistics of the cotton fruit dataset.

Class Name	Number of original images	Number of augmented images
Bacterial Blight	250	1000
Curl Virus	431	1000
Herbicide Growth Damage	280	1000
Leaf Hopper Jassids	225	1000
Leaf Redding	578	1000
Leaf Variegation	116	1000
Healthy Leaf	257	1000
**Total**	**2137**	**7000**

### 4.6 Model Validation

Inception V3, another prominent architecture in image classification, has garnered attention for its exceptional performance and computational efficiency. Introduced as an evolution of the Inception architecture, Inception V3 incorporates innovative features such as inception modules with factorized convolutions and aggressive dimensionality reduction]. These design choices enable the model to capture intricate patterns in images while minimizing computational overhead [18]. Inception V3 has demonstrated impressive results on benchmark datasets like ImageNet, showcasing its ability to accurately classify images across a broad spectrum of categories. Its efficient design facilitates rapid inference, making it suitable for real-time applications where quick decision-making is essential. With its blend of accuracy and efficiency, Inception V3 has established itself as a cornerstone in image classification, empowering developers to create robust AI solutions for a variety of applications [19], spanning from mobile applications to embedded systems and beyond.

In this study, we conducted a comprehensive evaluation of the Inception V3 architecture for the classification of cotton diseases. The dataset consists of images belonging to seven different classes representing various cotton diseases. We used 80 % of the data for training and 20 % for validation. To train the model, we utilized data augmentation techniques. These techniques help increase the diversity of the training data and improve the model's ability to generalize to unseen examples. The model was trained for 30 epochs using a batch size of 32, with a learning rate scheduler applied to dynamically adjust the learning rate during training. To gauge the effectiveness of the models, a range of evaluation metrics was utilized, encompassing accuracy, precision, recall, and F1 score (Eqs. (1) to (4)) [18]. These metrics offer a comprehensive assessment of the models' performance, providing insights into their classification capabilities from different perspectives. Also, we monitored the training progress using both training and validation datasets, with TensorBoard used for visualization and analysis of training metrics.

(1) $$\text{Accuracy}\;=\;\frac{\text{True}\;\text{Positive}+\text{True}\;\text{Negative}}{\text{True}\;\text{Positive}+\text{True}\;\text{Negative}+\text{False}\;\text{Positive}+\text{False}\;\text{Negative}}$$

(2) $$\text{Precision}\;=\;\frac{\text{True}\;\text{Positive}}{\text{True}\;\text{Positive}+\text{False}\;\text{Positive}}$$

(3) $$\text{Recall}=\;\frac{\text{True}\;\text{Positive}}{\text{True}\;\text{Positive}+\text{False}\;\text{Negative}}$$

(4) $$F1\;\text{Score}=2\times \frac{\text{Recall}\;\times \text{Precision}}{\text{Recall}\;\pm \text{Precision}}$$

The confusion matrix serves as a critical tool for evaluating the performance of machine learning models, providing insights into their classification accuracy and error patterns [20]. In our research, we utilized the confusion matrix to analyze the performance of our model for classifying cotton diseases. The matrix visually represents the model's predictions against the ground truth labels across different disease categories.

**Fig. 6:**
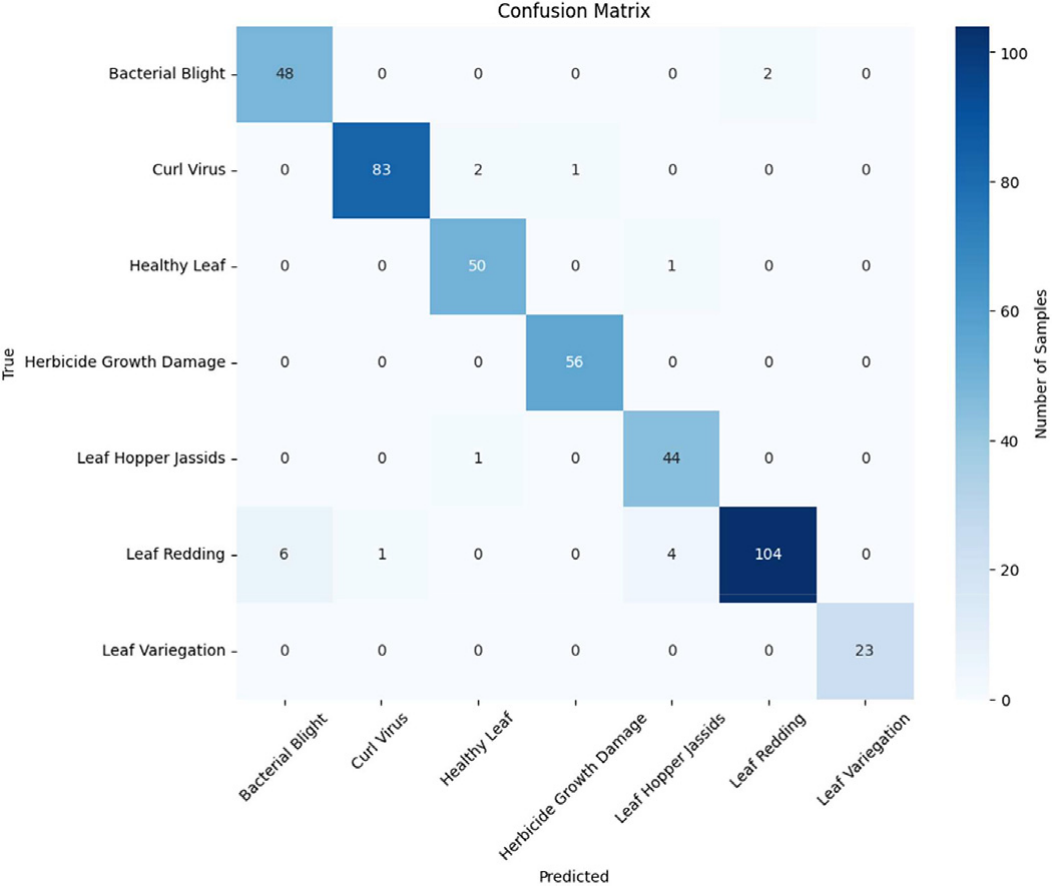
Confusion matrix for Inception V3

The Loss and Accuracy graphs in Fig. 6 showcase the performance of the Inception V3 model for cotton disease classification across 30 epochs. The Accuracy graph displays the model's learning trajectory, with the blue line indicating training accuracy and the orange line representing validation accuracy. It highlights the model's progress over time and any disparities between training and validation accuracy. Meanwhile, the Loss graph depicts the model's loss reduction throughout training, with the blue line representing training loss and the orange line indicating validation loss. The graphs provide insights into the model's training dynamics and performance stability.

**Fig. 7:**
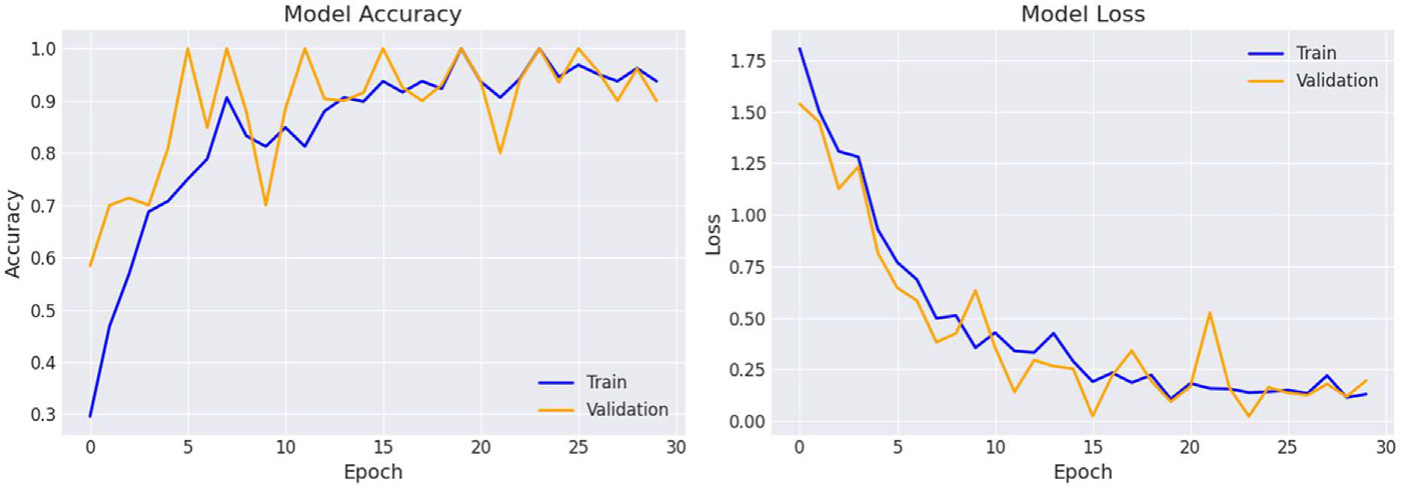
Accuracy and loss graph for Inception V3.

Following our evaluation with this dataset, the model exhibited remarkable efficiency, achieving an impressive accuracy rate of 96.03 %, as depicted in Table 4. This outcome underscores the model's robust ability to effectively distinguish between various types of cotton diseases. The high accuracy achieved highlights the reliability and potential applicability of the model in real-world agricultural scenarios (Fig. 7).

**Table 4:** Model Evaluation Metrics Classification report and Accuracy for Inception V3.

Model	Class Name	Precision	Recall	F1-score	Accuracy
Inception V3	Bacterial Blight of Cotton	0.89	0.96	0.92	96.03 %
	Curl Virus	0.99	0.97	0.98	
	Healthy Leaf	0.94	0.98	0.96	
	Herbicide Growth Damage	0.98	1.00	0.98	
	Leaf Hopper Jassids	0.90	0.98	0.94	
	Leaf Redding	0.98	0.90	0.94	
	Leaf Variegation	1.00	1.00	1.00

Our study presents a comprehensive analysis of cotton disease classification using state-of-the-art deep learning techniques. Leveraging a dataset meticulously curated for this purpose, we trained and evaluated several convolutional neural network (CNN) models, including Inception V3, on the task of identifying various diseases and pests affecting cotton plants. Our results demonstrate the efficacy of these models, with Inception V3 exhibiting particularly promising performance, achieving an impressive accuracy rate of 96.03 %. The robustness of the models in distinguishing between different cotton diseases underscores their potential for practical deployment in agricultural settings, aiding farmers in early detection and mitigation strategies. Furthermore, our exploration of data augmentation techniques and evaluation metrics provides valuable insights into enhancing model performance and interpretability. Overall, this study contributes to the growing body of research in precision agriculture, offering practical solutions for crop health monitoring and management.

## Limitations

Our study is the lack of samples collected from countries other than Bangladesh, which may restrict the dataset's representativeness and generalizability to a broader geographical context.

## Ethics Statement

The research conducted for this paper did not involve any experiments or studies with humans or animals as subjects. The dataset used consists solely of images obtained by the authors, adhering to ethical guidelines. All consulted datasets are publicly accessible, and proper citation guidelines have been followed.

## CRediT authorship contribution statement

**Prayma Bishshash:** Conceptualization, Methodology, Writing – original draft, Data curation. **Asraful Sharker Nirob:** Conceptualization, Visualization, Data curation, Writing – original draft. **Habibur Shikder:** Writing – original draft, Validation, Data curation. **Afjal Hossan Sarower:** Supervision, Formal analysis, Writing – review & editing. **Touhid Bhuiyan:** Writing – review & editing. **Sheak Rashed Haider Noori:** Writing – review & editing.

## Data Availability

SAR-CLD-2024: A Comprehensive Dataset for Cotton Leaf Disease Detection (Original data) (Mendeley Data).
